# Maternal embryonic leucine zipper kinase: A novel biomarker and a potential therapeutic target of cervical cancer

**DOI:** 10.1002/cam4.1816

**Published:** 2018-10-18

**Authors:** Juan Wang, Yamei Wang, Fangrong Shen, Yanting Xu, Yinghui Zhang, Xinwei Zou, Jinhua Zhou, Youguo Chen

**Affiliations:** ^1^ Department of Obstetrics and Gynecology The First Hospital Affiliated Soochow University Suzhou China

**Keywords:** apoptosis, cervical cancer, chemoradiotherapy, DNA damage

## Abstract

Maternal embryo leucine zipper kinase (MELK) is highly expressed in a variety of malignant tumors and involved in cell cycle regulation, cell proliferation, apoptosis, tumor formation etc However, the biological effects of MELK in cervical cancer are still uninvestigated. This study aimed to explore the expression of MELK in cervical cancer, as well as its effects on the proliferation, apoptosis, DNA damage repair on cervical cancer cell line in vitro and to provide novel ideas for further improving the clinical efficacy of cervical cancer. Immunohistochemistry, Western blot, RT‐qPCR, CCK8, and immunofluorescence techniques were used to detect the expression of MELK in cervical cancer tissues, paracancerous tissues, and cervical cancer cell lines. Several cervical cancer cell lines were treated with MELK knockdown by siRNA and MELK selective inhibitor OTSSP167. The effects on proliferation, apoptosis, and colony formation capacity, and tumor cell DNA damage repair‐related factor were detected in cell lines. Our data showed that the high expression rate of MELK in cervical cancer patients was 56.92%. MELK expression in cervical cancer samples was significantly higher than that in paraneoplastic tissues. Highly expressed MELK correlated with the cervical histopathological grading and greatly increased with the cervical histopathological grading, from normal cervix and cervical intraepithelial neoplasia to cervical cancer. Moreover, the abnormal expression of MELK was related to cervical cancer metastasis at early stage. The knockdown of MELK with siRNA and OTSSP167 had strong inhibition effects on the proliferation, apoptosis, and colony formation of cervical cancer cells. MELK knockdown could also aggravate the DNA damage of cervical cancer cells possibly by homologous recombination repair pathway. Therefore, MELK may be a predicting marker of poor prognosis of cervical cancer and may also be a new therapeutic target for cervical cancer, providing ideas for improving the therapeutic effect of cervical cancer.

## 1 INTRODUCTION

Cervical cancer is one of the most common malignant tumors in the female reproductive system, imposing harm to women's health. According to the WHO reporting, there are about 530 000 new cases of cervical cancer each year in the worldwide. And the annual mortality is about 260 000 cases, of which 90% are from developing countries.^1^ With effective screening and vaccination against human papillomavirus (HPV) in high‐risk populations, significant progress has been made in the early diagnosis and prevention of cervical cancer.^2^ Radical surgery and adjuvant chemotherapy are main treatments for early or locally advanced cervical cancer in patients.^3^ However, the 5‐year survival rate is greatly reduced because of metastasis and recurrence of cervical cancer. The retreatments are often unresponsive in these patients. During the past 30 years, survival rate has not been improved in advanced and recurrent cervical cancers.^4^, ^5^ Therefore, searching a new therapeutic target of cervical cancer is critically needed to enhance the sensitivity of chemoradiotherapy, reduce recurrence and metastasis after chemotherapy and sequentially improve the disease prognosis.^6^, ^7^, ^8^

Maternal embryonic leucine zipper kinase (MELK) is a kind of cell cycle dependent conserved serine/threonine protein kinase, at chromosome 9p13.2. Unlike other members of the family, MELK is not related to cellular energy metabolism balance.^9^ MELK is highly expressed in a wide range of malignant tumors, including hepatocellular carcinoma,^10^ breast cancer,^11^ melanoma,^12^ acute leukemia,^13^ ovarian cancer,^14^ neuroblastoma^15^ and myeloma.^16^ It is actively involved in cell cycle regulation, proliferation, apoptosis, and tumor formation.^9^^,^ 17 However, the role of MELK in cervical cancer has not been elucidated. The aim of this study is to investigate the expression of MELK in cervical cancer, as well as the effect on cancer cell proliferation, apoptosis, and DNA damage repair, so as to provide new ideas for further clinical improvement of cervical cancer.

## 2 MATERIALS AND METHODS

### 2.1 Bioinformatics analysis

Maternal embryo leucine zipper kinase gene expression was analyzed using microarray gene datasets from Oncomine database (https://www.oncomine.org). The differential expression of MELK was determined between cervical cancer and the normal cervix, by defining the type of cancer as cervical cancer. The data type was mRNA, and the type of analysis was cancer compared to the normal. The Oncomine algorithm was used to make a comparative statistical analysis.

### 2.2 Subjects

Patients with cervical intraepithelial neoplasia (CIN) and cervical cancer were included from December 2016 to October 2017 at the Department of Gynecology affiliated Soochow University. The control group had other benign tumors of the female reproductive system treated by total hysterectomy, and post‐operative cervix pathology was normal. The patients’ informed consents were obtained, and the study was approved by the institution ethics committee (2018LS065).

### 2.3 Immunohistochemistry staining assay

After the specimens were fixed by formaldehyde, ethanol was then used for dehydration to produce paraffin‐embedded blocks. Specimens were sliced into 4‐mm thick tissue sections. They were baked for 30 minutes at 60°C in the oven. After being dewaxed and hydrated, the tissue sections were washed with PBS solution and immersed in the 0.01 mol/L citrate buffer (pH 6). The sections were pressed for 3 minutes and left in natural cooling. When being taken out, one drop of peroxidase blocking agent was added to block the activity of endogenous peroxidase. The tissue sections were washed with PBS solution again and 50 µL diluted anti‐MELK[2G2] antibody (the optimum concentration was 1:200; Abcam company, Cambridge, UK) was added, then incubated in 4°C refrigerator overnight. The next day, after PBS washing, 50 µL second antibody (Shanghai Gene Technology Company, Shanghai, China) was added, and tissue sections were incubated for 30 minutes at room temperature. The subsequent procedures included PBS washing, brown staining with glucose oxidase‐DAB, redyeing of hematoxylin solution, dehydration, being transparent, neutral resin sealing, and finally further microscopic observation. The immunohistochemical staining results were assigned a mean score considering both the intensity of staining and the proportion of tumor cells with an unequivocal positive reaction. Each section was independently assessed by two pathologists without prior knowledge of patient data. Positive reactions were defined as those showing brown signals in the cell cytoplasm. A staining index (values, 0‐12) was determined by multiplying the score for staining intensity with the score for positive area. The intensity was scored as follows: 0, negative; 1, weak; 2, moderate; and 3, strong. The frequency of positive cells was defined as follows: 0, <5%; 1, 5%‐25%; 2, 26%‐50%; 3, 51%‐75%; and 4, more than 75%. When the staining was heterogeneous, we scored it as follows: each component was scored independently and summed for the results. For example, a specimen containing 75% tumor cells with moderate intensity (3 × 2 = 6) and another 25% tumor cells with weak intensity (1 × 1 = 1) received a final score of 6 + 1 = 7. For statistical analysis, score of 0‐7 was considered low expression and scores of 8‐12 considered high expression.

### 2.4 Cervical cancer cell lines, siRNA‐mediated MELK knockdown and its inhibitor OTSSP167 application

Human cervical cancer cell lines: SiHa cells, HeLa cells, CasKi cells, and C33A cells were purchased from the Shanghai Academy of life sciences (Chinese Academy of Sciences, Shanghai, China). SiHa cells, CasKi cells, and C33A cells were cultured in RPMI‐1640 (HyClone, Logan, UT, USA) supplemented with 10% fetal bovine serum (Biosera, Nuaille, France) and 1% penicillin‐streptomycin (Beyotime, Shanghai, China). HeLa cells were cultured in the DMEM culture medium, containing 10% fetal bovine serum and 1% penicillin‐streptomycin. The selective inhibitor of MELK is OTSSP167 (Selleck, Houston, TX, USA).The chemically synthesized MELK specific double stranded siRNA targeting sequences were from Shanghai Aibos Biology Company, Shanghai, China:

siRNA#1: Sense 5′‐GACUAAAGCUUCACUAUAACG‐3,

Anti sense 5′‐UUAUAGUGAAGCUUUAGUCUU‐3;

siRNA#2: Sense 5′‐GGAUCAUGCAAGAUUACAACU‐3,

Anti sense 5′‐UUGUAAUCUUGCAUGAUCCAG‐3.

### 2.5 Western blot analysis

Western blot was performed according to standard protocols. The total protein was extracted firstly. BCA Kit (Jiangsu Biyuntian Biotechnology Institute, Nantong, China) was used to detect protein concentration. Then the process included preparation of SDS‐PAGE gel, protein electrophoresis, protein electro‐transferring, and Western blot analysis.

### 2.6 RT‐qPCR analysis

Maternal embryo leucine zipper kinase RNA in cervical cancer tissues, paracancerous tissues, and four cervical cancer cell lines was detected by quantitative real‐time polymerase chain reaction. Total RNA was firstly extracted with TRIzol reagent (Invitrogen, Carlsbad, CA, USA). The beta‐actin was used as an internal reference. For mRNA expression analysis, RevertAid First Strand cDNA Synthesis Kits (Thermo Fisher Scientific, Carlsbad, CA, USA) were used for cDNA synthesis. The reverse transcriptase cDNA worked as a template. The primer sequences were: MELK forward: 5′‐GCT GCA AGG TAT AAT TGA TGGA‐3′, reverse: 5′‐CAG TAA CAT AAT GAC AGA TGGGC‐3′; β‐actin forward: 5′‐TGACGTGGACATCCGCAAAG‐3′, reverse: 5′‐CTGGAAGGTGGACAGCGAGG‐3′. RT‐qPCR was performed using Power SYBR Green PCR Master Mix (Takara, Otsu, Shiga, Japan) according to the manufacturer's instructions. The reactions were performed in CFX96 (Bio‐Rad company, Shanghai, China) and the reaction mix was incubated at 95°C for 10 minutes, followed by 40 cycles at 95°C for 15 seconds, 60°C for 30 seconds, and 72°C for 30 seconds. Data analyses were performed using the 2^−ΔΔCt^ method (Tables S1, S2).

### 2.7 Detection of cell proliferation by Cell‐Counting Kit‐8 assay

The cervical cancer cell lines C33A and CasKi were, respectively, divided into three subgroups: (a) the control, (b) siRNA#1 transfection, (c) siRNA#2 transfection. Each subgroup had three repeating holes. The absorbance of 450 nm was detected by enzyme labeling at 24, 48, 72, 96, and 120 hours after transfection. In the control subgroup, cell growth curve was drawn after zero adjustment. The cervical cancer cell lines C33A, SiHa, and HeLa were, respectively, divided into five to six subgroups: (a) 0 nmol/L, (b) 2.5 nmol/L, (c) 5 nmol/L, (d) 10 nmol/L, (e) 20 nmol/L, (f) 40 nmol/L. Each subgroup had three repeating holes. The absorbance of 450 nm was detected by enzyme labeling at 24, 48, 72, 96, and 120 hours after OTSSP167. In the control subgroup, cell growth curve was drawn after zero adjustment.

### 2.8 Cloning formation test to detect cell growth

The cervical cancer cell lines C33A and HeLa were divided into three subgroups: (a) the control; (b) siRNA#1 transfection; (c) siRNA#2 transfection. The cervical cancer cell lines C33A and HeLa were, respectively, divided into three subgroups with different OTSSP167 concentrations: (a) 0 nmol/L, (b) 10 nmol/L, (c) 20 nmol/L; and (a) 0 nmol/L, (b) 20 nmol/L, (c) 40 nmol/L. Forty‐eight hours after transfection or OTSSP167 suppression, a total of 500 cells per pore were inoculated with three repeated holes in each subgroup. After 7‐10 days, cell lines were stained with crystal violet for 30 minutes and observed by the microscope.

### 2.9 Immunofluorescence observation of DNA damage foci

The cervical cancer cell lines C33A and HeLa were divided into three subgroups: (a) the control, (b) siRNA#1 transfection, (c) siRNA#2 transfection. The cervical cancer cells were inoculated with the density of the 5 × 10^4^ cells/hole in the 24‐hole cell plate with small round slide. The siRNA was transfected for 48 hours. The different experimental subgroups were treated with different interventions. The cells were washed with precooled PBS for three times. About 4% polyformaldehyde was added for fixation. With being washed by the ice PBS two times, the cells were incubated with 0.5% triton for 10 minutes, then washed again with PBS two times. Blocked in 5% BSA PBS solution at room temperature for 1 hour, the small round slides were taken out and inverted to the sealed membrane with rabbit anti‐γ‐H2AX antibody (Cell Signaling Technology Company, Boston, MA, USA). Incubated overnight, the slides were then placed in the 12‐hole plates. After being washed with PBS, the small slides were inverted to the sealed membrane with the second antibody (R&D Company, Lorton, VA, USA), and the washing steps were the same as the above. In the dark place, the DAPI solution was used to dye the nucleus for 30 minutes, and the small slides were dealt with the anti‐fluorescent quenching agent. Finally, the slides were observed and photographed under the fluorescence microscope.

### 2.10 Statistical analysis

Each experiment was independently repeated three times or more. Data were presented as percentage and proportion. Statistical analysis was performed using Pearson's chi‐square and Fisher's exact tests. Partitions of chi‐square methods were applied for further comparison of two sample frequencies. Value of *P* < 0.05 (two‐sided) was considered as statistical significance. SPSS software version 22.0 (IBM, Armonk, NY, USA) was used for all the data analysis.

## 3 RESULTS

### 3.1 Clinical characteristics

A total of 120 cases, aged 30‐72 years, were included in this study: normal cervical tissue, n = 10; Grade CIN I, n = 12; Grade CIN II, n = 15; Grade CIN III, n = 18; and cervical cancer, n = 65 (including 28 cases of cervical cancerous and paracancerous paired tissues). Histopathology of cervical cancer identified squamous cell carcinoma (n = 53), adenocarcinoma (n = 9), and adenosquamous carcinoma (n = 3). According to the Cervical Cancer Staging System of International Federation of Gynecology and Obstetrics in 2014, there were 50 patients at early stages (Ia, n = 3; Ib1, n = 19; Ib2, n = 3; IIa, n = 25), of which 23 patients had intravascular and/or lymphatic metastasis. The other 15 patients were at the advanced stages (IIb, n = 1; III, n = 10; IV, n = 4).

### 3.2 Overexpressed MELK in cervical cancer by bioinformatics analysis

**Figure 1:**
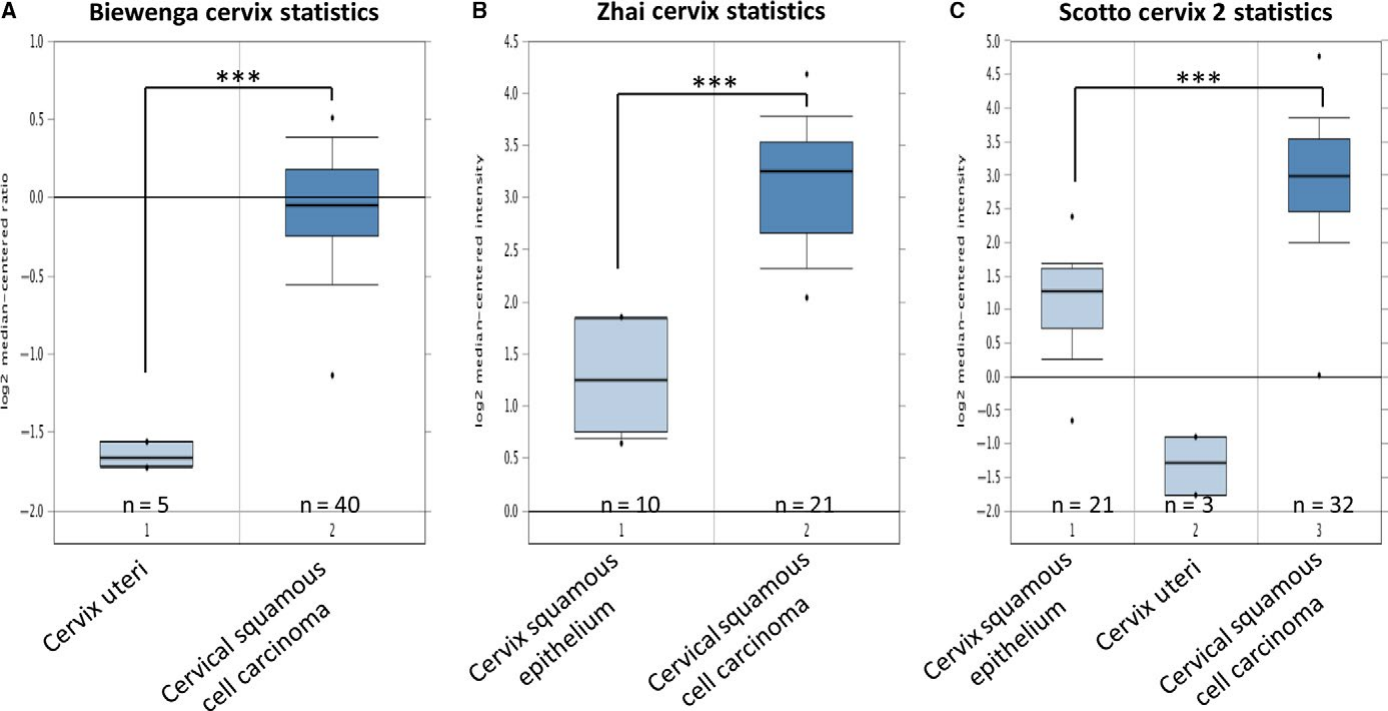
Maternal embryonic leucine zipper kinase (MELK) is differentially expressed in the datasets of human cervical cancer expression spectrum. Bioinformatics analysis was performed on Biewenga Cervix Statistics, Zhai Cervix Statistics, and Scotto Cervix two Statistics datasets. The expression level of MELK mRNA in cervical squamous cell carcinoma was significantly higher than in the corresponding normal cervical tissue (***P < 0.001)

In order to study the relationship between MELK and cervical cancer progression, differential expression of MELK mRNA in cervical cancer tissues and normal cervical tissues was determined by analyzing the Oncomine microarray gene expression datasets. Bioinformatics analysis of three datasets of Biewenga Cervix Statistics,^18^ Zhai Cervix Statistics,^19^ and Scotto Cervix 2 Statistics^20^ showed that the expression level of MELK in cervical cancer tissues was significantly higher than in normal cervical tissues (Figure 1A‐C).

### 3.3 The high expression level of MELK increased with the cervical histological grading

**Figure 2:**
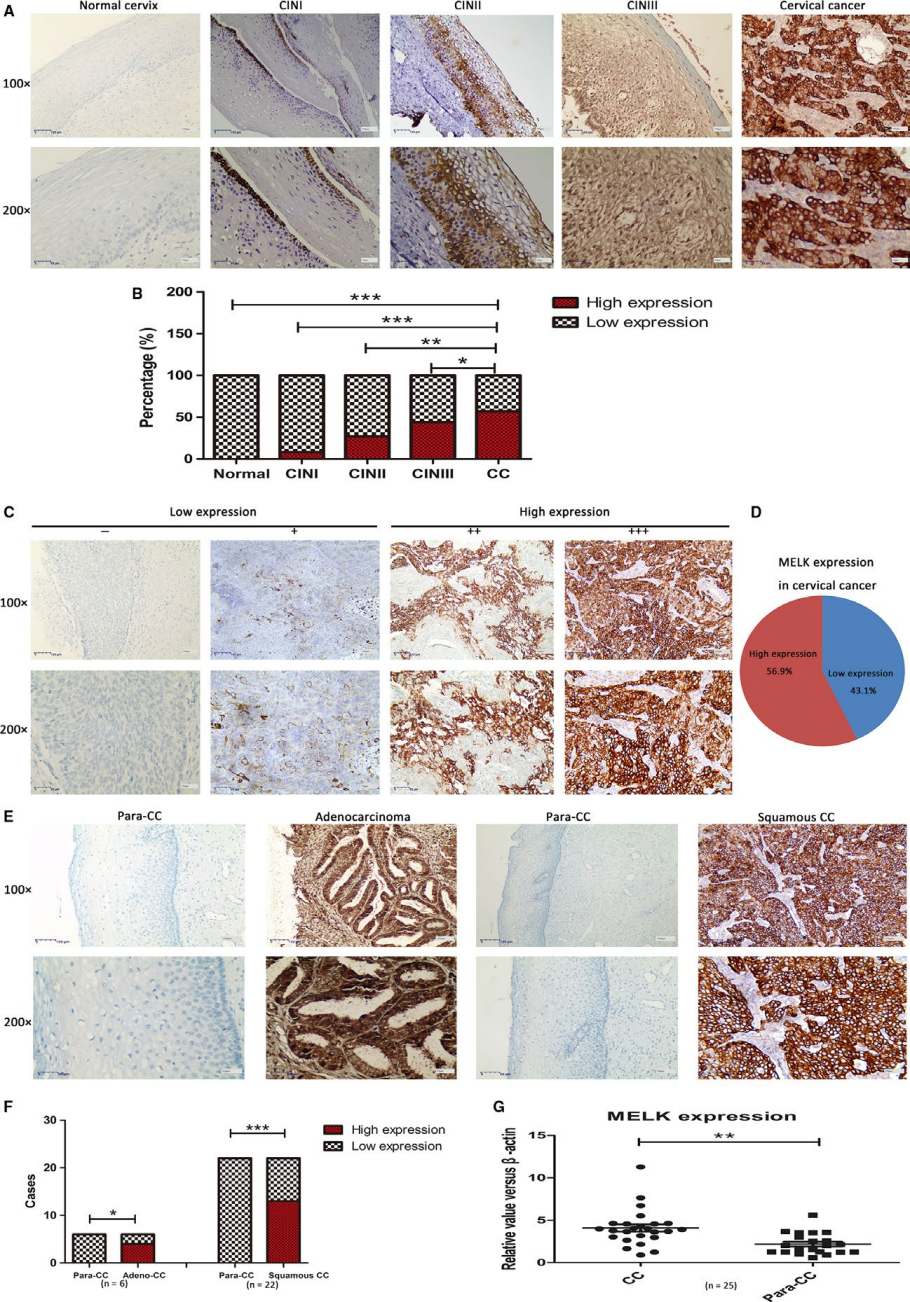
A, MELK expression in normal cervix, CIN I‐III (cervical intraepithelial neoplasia Grade I‐III), and cervical cancer by immunohistochemistry assay; B, MELK high expression rate was significantly more in cervical than that in normal cervix or CIN I‐III, in correlation with cervical disease grade; C, Positive reactions were defined as those showing brown signals in the cell cytoplasm. A staining index (values, 0‐12) was determined by multiplying the score for staining intensity with the score for positive area. The intensity was scored as follows: 0, negative; 1, weak; 2, moderate; and 3, strong. The frequency of positive cells was defined as follows: 0, <5%; 1, 5%‐25%; 2, 26%‐50%; 3, 51%‐75%; and 4, more than 75%. For statistical analysis, score of 0‐7 was considered low expression and scores of 8‐12 considered high expression. D, The high expression rate of MELK in cervical carcinoma (n = 65) was 56.9%; E, Paracancerous cervical adenocarcinoma tissues, cervical adenocarcinoma, paracancerous cervical squamous carcinoma tissues and squamous carcinoma from left to right. None MELK‐positive staining cells were detectable in paracancerous tissues, compared to large number of positive staining cells in carcinoma tissues. F, Immunohistochemistry showed that the high expression rate of MELK in cancer tissues was significantly more than in paired paracancerous tissues (n = 28); G, RT‐qPCR was used to detect mRNA in paired cancerous and paraneoplastic tissues (n = 25). The findings showed that the MELK mRNA expression in cervical cancer tissues was significantly higher than that of paracancerous tissues (*P < 0.05, **P < 0.005*,* ***P < 0.001)

To verify the abnormal expression of MELK gene during cervical pathological process, immunohistochemistry studies were conducted in normal cervix, CIN, and cervical cancer tissues. The results revealed that higher MELK expression was related to cervical abnormality severity. MELK expression was progressively increased from normal cervix, CINI, CINII, CINIII to cervical cancer (Figure 2A). MELK expression was low in all normal cervical tissues of 10 controls. The percentage of high expression MELK was, respectively, 8.33% (1/12) in CIN Grade I, 26.67% (4/15) in CIN Grade II, 44.44% (8/18) in CIN Grade III, and 56.92% (37/65) in cervical cancer patients (Figure 2B). MELK high expression was significantly more in cervical cancer than in the normal or any CIN (*P* < 0.01, Figure 2B, Table 1). A total of 65 cervical cancer tissue specimens were collected in this study. The immunohistochemical results showed that the high expression rate of MELK was 56.92% (37/65; Figure 2C,D).

**Table 1:** MELK expression by immunohistochemistry assay greatly increased with the cervical histopathological grading

Histopathological diagnosis	n	MELK expression (%)		*P*‐value
		Low	High	
Normal	10	10 (100.00)	0 (00.00)	<0.001
CINⅠ	12	11 (91.67)	1 (8.33)	
CINⅡ	15	11 (73.33)	4 (26.67)	
CINⅢ	18	10 (55.56)	8 (44.44)	
Cervical cancer	65	28 (43.08)	37 (56.92)	

CIN, cervical intraepithelial neoplasia; MELK, Maternal embryonic leucine zipper kinase.

### 3.4 The expression level of MELK in cervical cancer was significantly higher than that in paracancerous tissues

Immunohistochemistry indicated that the high expression of MELK in cervical cancer tissues was significantly more than that in the paracancerous tissues. In collected cancerous and the corresponding paraneoplastic tissues from 28 cervical cancer patients, 66.67% (4/6) of cervical adenocarcinoma patients and 59.09% (13/22) of squamous cell carcinoma patients displayed MELK high expression results. In this regard, no MELK high expression result was observed in all the corresponding paraneoplastic tissues (*P < 0.05, Figure 2E,F). RT‐qPCR technique was used to detect mRNA in 25 paired specimens of cancerous and paraneoplastic tissues. The findings revealed that the average expression level of MELK mRNA in cervical cancer tissues was significantly higher than that in paired adjacent tissues (*P* < 0.001, n = 25, Figure 2G).

### 3.5 Expression of MELK in cervical cancer cell lines

**Figure 3:**
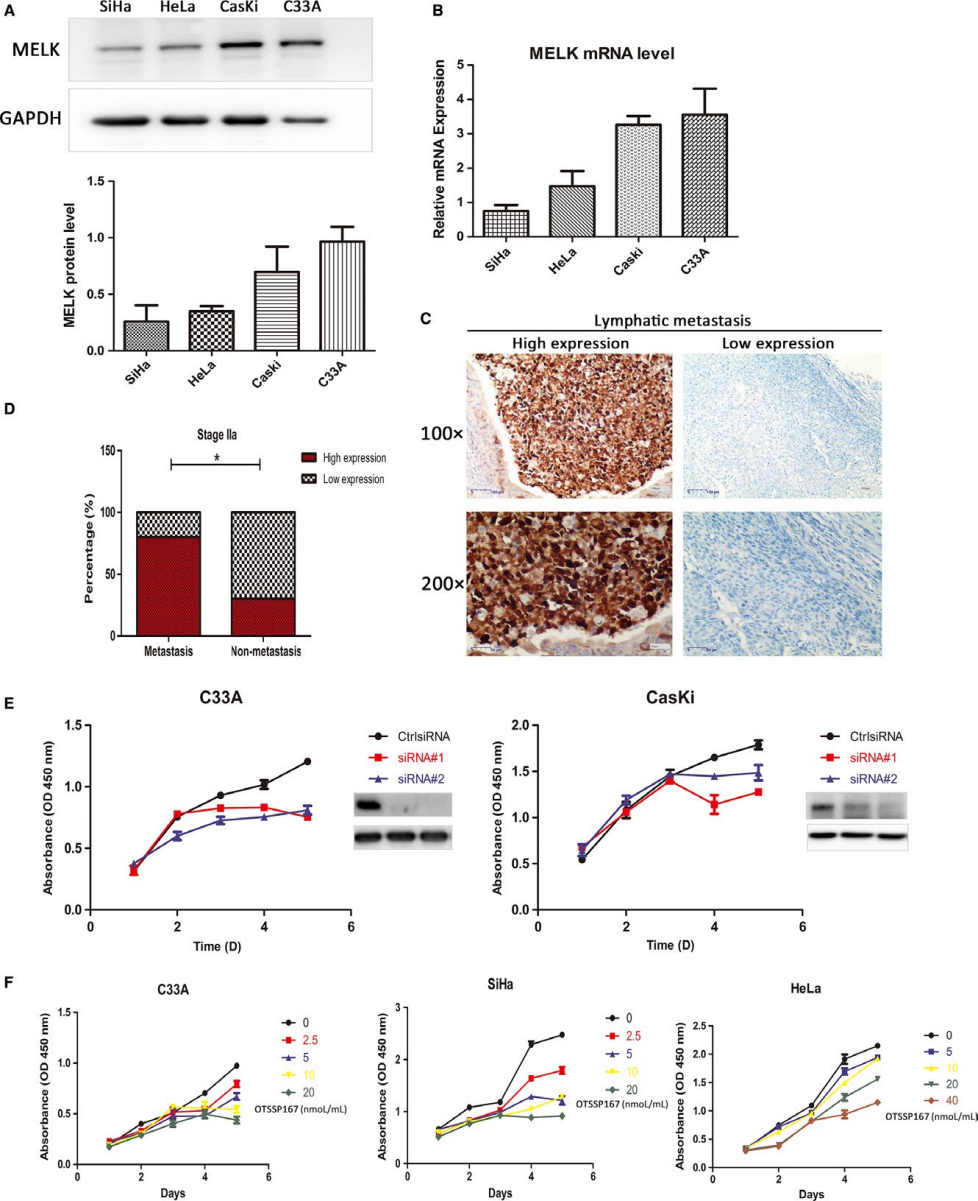
A, MELK was generally overexpressed in all four cervical cancer cell lines SiHa, HeLa, C33A and CasKi by Western blot; B, MELK mRNA was especially elevated in C33A and CasKi cell lines by RT‐qPCR; C, MELK expression was progressively increased in metastatic lymph nodes of cervical carcinoma patients by immunohistochemistry assay; D, At the stage IIa of cervical cancer, high expression of MELK was more in patients with metastasis compared to those without metastases; E, Transfection of cervical cancer cells C33A and CasKi with siRNA#1, siRNA#2, the results showed that the cell growth in MELK knockdown groups was slower during 0‐120 h, compared to the control; F, C33A, SiHa, and HeLa cells were treated by MELK inhibitor OTSSP167 within a certain concentration range (0‐40 nmol/L/mL). With the increase in OTSSP167 concentration, the inhibition rate of C33A, SiHa, and HeLa cells also was added. Ctrl siRNA was the control (*P < 0.05)

The expression levels of MELK in all four cervical cancer cell lines SiHa, HeLa, C33A, and CasKi were detected by Western blot and RT‐qPCR, as shown in Figure 3A. MELK was expressed in all four cervical cancer cell lines, especially in C33A and CasKi cells (Figure 3B).

### 3.6 Overexpressed MELK correlated with clinical pathological features

Maternal embryo leucine zipper kinase high expression was not strongly correlated with age (*P* = 0.544), serum squamous cell carcinoma antigen (*P* = 0.668), and histological staging (*P* = 0.456). Furthermore, there was no significant difference related to cervical cancer pathological types, in 53 cases of squamous cell carcinoma, nine cases of adenocarcinoma, and three cases of adenosquamous carcinoma (*P* = 0.602, Table 2.

**Table 2:** Correlation of MELK expression by immunohistochemistry with clinicopathological parameters in cervical cancer patients

Clinical parameters	n	MELK expression (%)		*P*‐value
		Low	High	
Age (years)				
≤50	35	13 (37.14)	22 (62.86)	0.544
>50	30	9 (30.00)	21 (70.00)	
Serum SCCA				
≤5	39	20 (51.28)	19 (48.72)	0.668
>5	22	10 (45.45)	12 (54.55)	
Unknown	4	1 (25.00)	3 (75.00)	
FIGO staging				
Ia	3	2 (66.67)	1 (33.33)	0.456
Ib1	19	4 (21.05)	15 (78.95)	
Ib2	3	1 (33.33)	2 (66.67)	
IIa	25	10 (40.00)	15 (60.00)	
IIb	1	1 (100.00)	0 (00.00)	
III	10	4 (40.00)	6 (60.00)	
IV	IV4	2 (50.00)	2 (50.00)	
Pathological type				
Squamous cell carcinoma	53	23 (43.40)	30 (56.60)	0.602
Adenocarcinoma	9	3 (33.33)	6 (66.67)	
Adenosquamous carcinoma	3	2 (66.67)	1 (33.33)	
Early tumor metastasis				
Stage Ib1				
Yes	8	0	8 (100.00)	0.103
No	11	4 (36.36)	7 (63.63)	
Stage IIa				
Yes	15	3 (20.00)	12 (80.00)	0.034*
No	10	7 (70.00)	3 (30.00)	
Tumor differentiation				
Medium‐low	38	12 (31.03)	26 (68.97)	0.100
High	27	14 (51.85)	13 (48.15)	
Early infiltration depth				
Stage Ib1				
Deep muscular layer	5	1 (20.00)	4 (80.00)	1.000
Superficial layer	14	4 (28.57)	10 (71.43)	
Stage IIa				
Deep muscular layer	16	5 (31.25)	11 (68.75)	0.397
Superficial layer	9	5 (55.56)	4 (44.44)	

MELK, Maternal embryonic leucine zipper kinase; SCCA, squamous cell carcinoma antigen; FIGO staging: the Cervical Cancer Staging System of International Federation of Gynecology and Obstetrics in 2014. Deep muscular layer: more than 1/2 myometrium being invaded by tumors. Superficial muscular layer: the invasion of less than and equal to 1/2 muscle layer.

a *P* < 0.05.

However, higher MELK expression was significantly associated with cervical cancer metastasis in the study. Tissue samples from 25 patients with Stage IIa cervical cancer were collected. Among them, 15 (60%, 15/25) patients presented pathological metastasis (lymphatic or intravascular metastases). The high expression rates of MELK were 80% (12/15) in cancer metastatic patients and 30% (3/10) in metastasis‐free patients, respectively (*P* = 0.034, Figure 3C,D). The results showed that the high expression rate of MELK was more in metastatic patients than in patients without metastases.

Maternal embryo leucine zipper kinase over expression was not significantly correlated with the depth of tumor invasion. Tissue samples of 25 patients with Stage IIa and 19 patients with Stage Ib1 cervical cancer were included. The invasion of pathological deep muscle layer was defined as more than 1/2 myometrium being invaded by tumors. The invasion of less than and equal to 1/2 muscle layer was considered as superficial muscle layer involvement. Twenty‐one patients (16 of Stage IIa and 5 of Stage Ib1) had deep muscle layer being invaded. And 23 patients (9 of Stage IIa and 14 of Stage Ib1) had superficial muscle layer involvement. The results suggested that the high expression rate of MELK was not significantly correlated with the depth of tumor invasion in early stage (*P* > 0.05, Table 2).

### 3.7 The effect of MELK knockdown and inhibitor OTSSP167 on the proliferation, apoptosis and colony formation of cervical cancer cells

**Figure 4:**
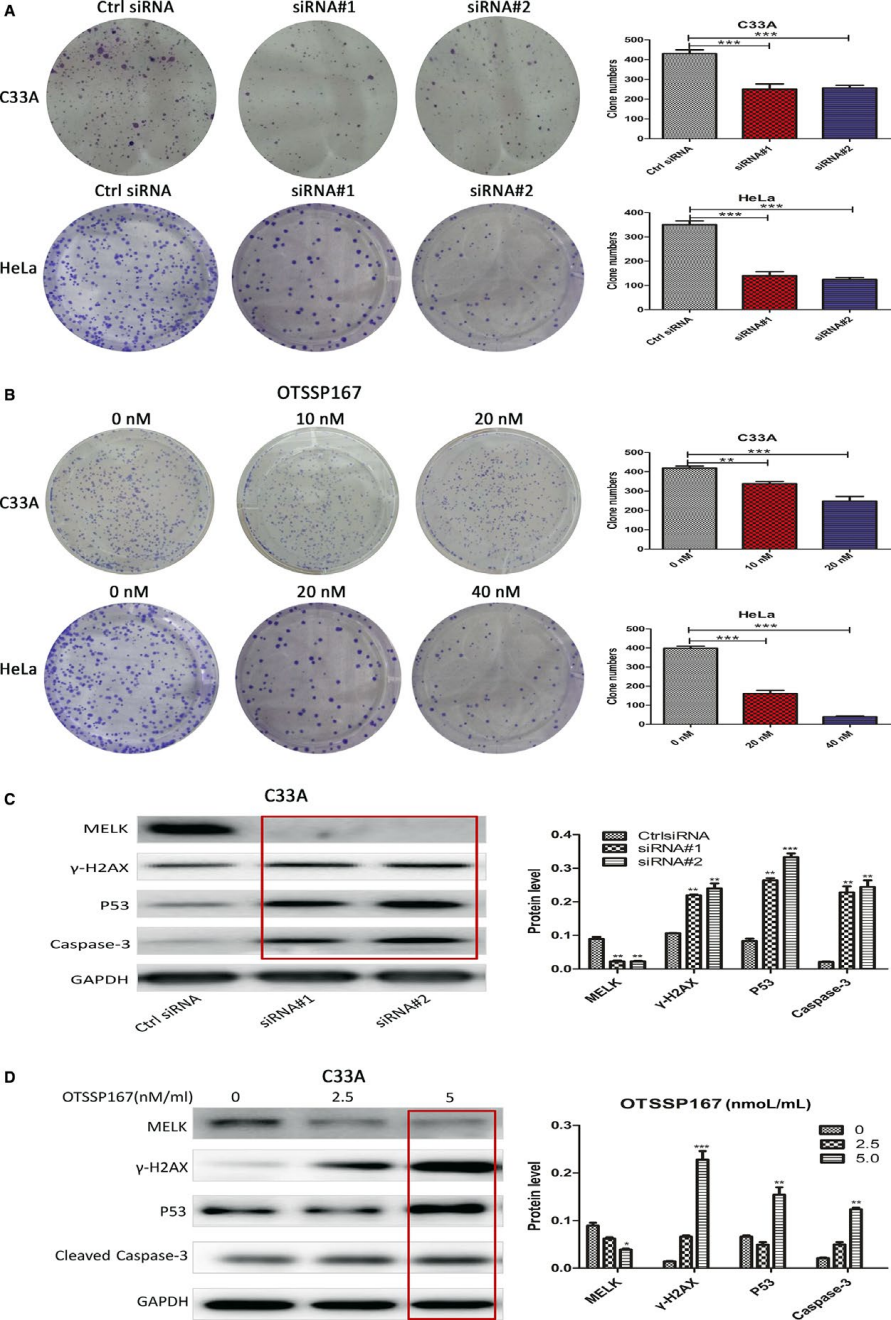
A, The cervical cancer cells C33A and HeLa were transfected with siRNA#1, siRNA#2. The results showed that MELK knockdown could inhibit the ability of cell C33A and HeLa colony formation; B, MELK inhibitor OTSSP167 was treated on cervical cancer C33A and HeLa cells, within a certain concentration range. As the concentration of OTSSP167 elevated, the inhibition on cells cloning formation was added; C, Cervical cancer cell C33A was transfected with siRNA#1, and siRNA#2, respectively. The expression of γ‐H2AX, P53, and cleaved caspase‐3 in the MELK knockdown group was significantly increased, compared to the control group; D, The C33A cell line was treated with the OTSSP167 within a certain concentration range. When the concentration of OTSSP167 added, the expression of γ‐H2AX, P53, cleaved caspase‐3 increased. Ctrl siRNA was the control (*P < 0.05, **P < 0.005, ***P < 0.001)

The cervical cancer cells C33A and CasKi were transfected with siRNA#1 and siRNA#2, respectively. The results showed that the cells with knockdown MELK by siRNAs grew slowly during 0‐120 hours, compared to the control. C33A, SiHa and HeLa cells were treated by OTSSP167 with different concentration, in a certain concentration range, the inhibitory rate of above cells increased as OTSSP167 concentration was raising (Figure 3E,F). Clone formation experiments suggested that both MELK knockdown and inhibitor OTSSP167 could inhibit the C33A and HeLa colony formation ability. Within a certain concentration range, the higher the concentration of OTSSP167, the greater inhibitory effect on the cells cloning, as shown in Figure 4A,B. The cervical cancer cell C33A was transfected with siRNA#1, and siRNA#2, respectively. The findings indicated that the expression of apoptosis‐related proteins P53 and cleaved caspase‐3 were significantly higher in MELK siRNA knockdown groups, in comparison to the control group. The C33A cell line was treated with the inhibitor OTSSP167. Within a certain concentration range, the cleaved caspase‐3 expression was elevated with the increase in the OTSSP167 concentration (Figure 4C,D).

### 3.8 MELK and DNA damage repair of cervical cancer cells

**Figure 5:**
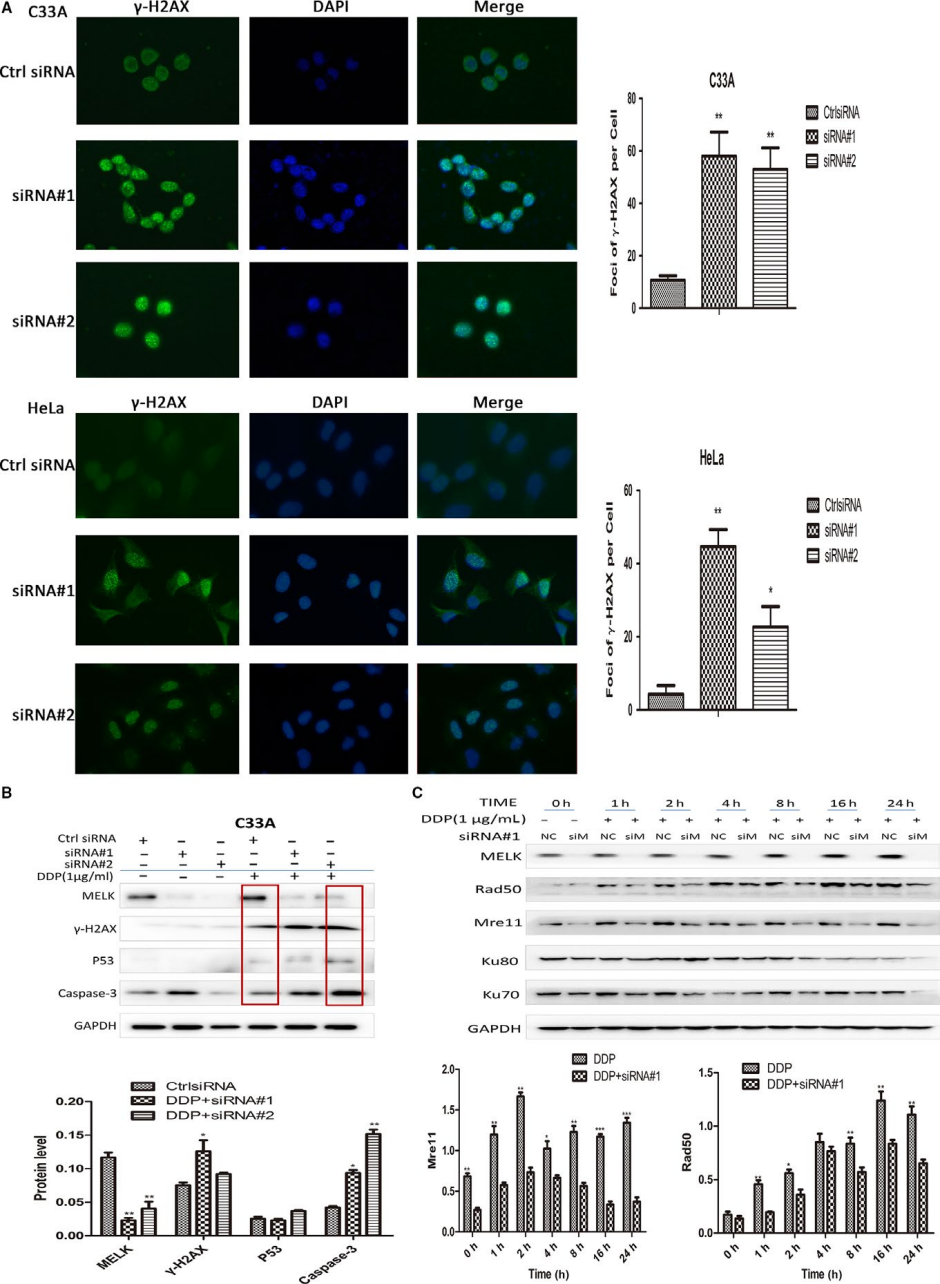
A, C33A and HeLa cell lines were transfected with siRNA#1, siRNA#2 to knock down MELK. Immunofluorescence was used to detect the formation of γ‐H2AX foci. The results showed that there were more foci formation in the HeLa and HeLa cells’ nucleus of MELK knockdown group, compared to the control group; B, The cervical cancer cells C33A were transfected with siRNA#1, siRNA#2, and then treated with DDP for 24 h. The expression of γ‐H2AX and cleaved caspase‐3 was significantly increased in MELK knockdown and DDP treated group than those in DDP alone group; C, The C33A cell was transfected with siRNA#1, and then treated with DDP (Cisplatin 1 µg/mL). The DNA damage repair proteins were detected by Western blot. The expression levels of Mre11 and Rad50 were significantly lower in MELK knockdown and DDP treated group than those in DDP alone group during 24 h; the expression levels of Ku70/80 were not significantly correlated with MELK knockdown and DDP treated group than those in DDP alone group; Ctrl siRNA was the control (*P < 0.05, **P < 0.005, ***P < 0.001)

Maternal embryo leucine zipper kinase knockdown could aggravate cervical cancer cells’ DNA damage. The cervical cancer cell C33A was transfected with siRNA#1, siRNA#2, or treated with OTSSP167 (0‐5 nmol/L/mL). γ‐H2AX, a marker protein of DNA damage‐breaking point, was further investigated. Western blot assay showed that the expression of γ‐H2AX was elevated in siRNA‐mediated MELK knockdown subgroups compared to the control subgroup (Figure 4C). Moreover, γ‐H2AX in C33A cells treated by OTSSP167 was greatly increased with OTSSP167 different concentration (Figure 4D). C33A and HeLa cells were transfected with siRNA#1 and siRNA#2, respectively. Cell immunofluorescence was used to detect γ‐H2AX foci formation. The results demonstrated that there were more foci formation in siRNA‐mediated MELK knockdown C33A and HeLa cells (Figure 5A). C33A cells were transfected with siRNA#1, siRNA#2, and then treated with Cisplatin (DDP). Western blot showed that the expression of γ‐H2AX of C33A cells in the groups with siRNA‐mediated MELK knockdown and treated by DDP group was increased, compared to DDP alone group (Figure 5B). At the same time, elevated caspase‐3 was observed in C33A cells treated by DDP combined with siRNA‐mediated MELK knockdown (Figure 5B).

Maternal embryo leucine zipper kinase mediated DNA damage repair of cervical cancer cells through homologous recombination pathway possibly. The cervical cancer cell C33A was transfected with siRNA#1, and then treated with DDP. Homologous recombination and non‐homologous end joining repair proteins involved in DNA damage were detected by Western blot. The results indicated that the expression levels of Mre11 and Rad50 were significantly lower in the group with MELK knockdown combined with DDP than those in the DDP alone group, the expression levels of Ku70 and Ku80 were not significantly correlated with MELK knockdown and DDP treated group than those in DDP alone group (Figure 5C).

## 4 DISCUSSION

Maternal embryo leucine zipper kinase is highly expressed in a wide range of cancer and is associated with the degree of malignancy of the tumor.^21^^,^ 17 This study confirmed that MELK mRNA and protein levels were up‐regulated in four cervical cancer cell lines. MELK was highly expressed in clinical specimens of cervical cancer patients and the high expression rate was 56.92%. However, there was no or low expression of MELK in normal cervical and paracancerous tissues of humans. MELK expression elevated with progression and severity of cervical diseases from the normal cervix, CINI, CINII, CINIII to cervical cancer. It suggests that MELK may play an important role in the oncogenic formation and progression of cervical cancer.

Over expression of MELK is associated with a poor prognosis in several malignancies.^17^, ^22^ In breast and gastric cancers, the expression of MELK correlated with the metastasis and recurrence of tumors. It is a novel marker for predicting cancer outcomes and a potential therapeutic target for some cancers.^23^^,^ 24 Interestingly, we also found similar effects of MELK on cervical cancers in the present study. In the same stage of IIa, the high expression rate of MELK in patients with lymphatic or vascular metastases was significantly more than that without metastasis. Thus, MELK may affect the biological behavior of cervical cancer.

OTSSP167 is a selective small‐molecule inhibitor against MELK, developed in 2012 and has been verified to inhibit proliferation in several tumor cells from different sources.^25^, ^26^ OTSSP167 has been used in healthy volunteers to assess its safety in Australia. The treatment for various solid tumors is currently being evaluated in clinical studies. Nevertheless, cervical tumors are not included.^27^ Kohler et al^14^ found that the use of MELK inhibitor OTSSP167 can induce G2/M cell cycle arrest, inhibit proliferation and colony formation, and induce apoptosis of HGSOC cell lines. Another two studies reported that MELK expression is associated with tumor cell mitosis^28^ and promotes tumor cell proliferation.^29^ MELK also plays an important role in the P53‐P21 apoptotic pathway.^30^ Based on the above findings, we used different concentrations of inhibitor OTSSP167 and siRNA knockdown MELK to detect cervical cell proliferation, colony formation ability, and apoptosis‐related proteins P53, cleaved caspase‐3. The results showed that MELK could significantly affect a variety of cervical cancer cell lines proliferation, colony formation ability and promote cell senescence and apoptosis. Within a certain concentration range, the increase in inhibitor OTSSP167 concentration accelerated cervical cancer cell lines’ proliferation inhibition rate, decreased cloning ability, and promoted cell senescence and apoptosis.

Inhibitors of tumor DNA damage repair pathways can induce tumor DNA damage and increase the efficacy of chemoradiotherapy.^31^, ^32^, ^33^ Targeting MELK function through the inhibitor MELK‐T1 can reduce the DNA damage tolerance threshold and make the tumor sensitive to DNA damaging agents or radiation therapy.^21^ MELK can increase the resistance to the radiation and 5‐FU treatments in colorectal cancer cells.^34^ MELK inhibition may be a novel and favorable strategy for chemoradiotherapy in cancers. In this report, knockdown of MELK transfected with siRNA caused DNA damage of cervical cancer cells. Furthermore, MELK knockdown combined with DDP treatment could aggravate the extent of DNA damage in the cells. Our findings in cervical cancer validated that the inhibition of MELK affected the cancer cells’ DNA damage repair. Non‐homologous end joining and homologous recombination repair are two important ways to repair DNA damage caused by platinum anticancer drugs and radiotherapy.^35^ MELK influences the extent and duration of DNA damage in triple‐negative breast tumors (negative estrogen receptor, progesterone receptor, and HER2) and plays a role in DNA double‐strand break repair.^11^ Homologous recombination repair depends on recruitment of the injury sites from MRN complex (a complex of three proteins, Mre11, Rad50, and NBS1) at the early stage.^36^^,^ 37 Our data indicated siRNA knockdown of MELK combined with DDP in cervical cancer cell significantly reduced the expression levels of Mre11 and Rad50, compared to DDP alone group. These findings demonstrated that MELK may repair DNA damage by the homologous recombination pathway. Unfortunately, this study did not find the evidence that MELK participated in non‐homologous end joining repair pathway.

In summary, MELK is highly expressed in cervical cancer and correlates with tumor metastasis. MELK is positively involved in cervical cancer cell proliferation, apoptosis, colony formation, and DNA damage repair. Therefore, MELK may be a novel biomarker predicting the poor prognosis of cervical cancer and a promising therapeutic target for the disease.

## CONFLICT OF INTEREST

None declared.
