# Supplementary material for: Maternal embryonic leucine zipper kinase: A novel biomarker and a potential therapeutic target of cervical cancer
Source: Cancer Med. 2018 Oct 18;7(11):5665–78. doi: 10.1002/cam4.1816 (PMC6246930; doi:10.1002/cam4.1816)
Supplement: Supplementary file 1 [file CAM4-7-5665-s001.docx]

**Reverse transcription for cDNA synthesis**

1) Reverse transcription according to the following table:

| Total RNA | 1ug |
| --- | --- |
| 5xPrimeScript RT Master Mix | 4ul |
| RNase Free dH2O | To 20ul |
| Total | 20ul |

2) Reaction under the following conditions:

| 37℃ | 15min |
| --- | --- |
| 85℃ | 5s |
| 4℃ | Hold |

cDNA product stored in - 20℃ refrigerator.

**Real-Time PCR Reaction**

1) Reaction system as followed:

| cDNA | 1ul |
| --- | --- |
| Primer（10uM） | 1ul each |
| 2xSYBR GREEN | 10ul |
| RNase Free dH2O | 7ul |
| Total | 20ul |

2）Real-Time reaction under the following conditions:

The reaction mixture was incubated at 50°C for 2min, denaturation at 95°C for 10 min, followed by 40 cycles at 95°C for 15s, 60°C for 30s and 72°C for 30s. Data analyses were performed using the 2^−ΔΔCt^ method.
